# The CXCL Family Contributes to Immunosuppressive Microenvironment in Gliomas and Assists in Gliomas Chemotherapy

**DOI:** 10.3389/fimmu.2021.731751

**Published:** 2021-09-13

**Authors:** Zeyu Wang, Yuze Liu, Yuyao Mo, Hao Zhang, Ziyu Dai, Xun Zhang, Weijie Ye, Hui Cao, Zhixiong Liu, Quan Cheng

**Affiliations:** ^1^Department of Neurosurgery, Xiangya Hospital, Central South University, Changsha, China; ^2^Clinic Medicine of 5-Year Program, Xiangya School of Medicine, Central South University, Changsha, China; ^3^Department of Clinical Pharmacology, Xiangya Hospital, Central South University, Changsha, China; ^4^Department of Psychiatry, The Second People’s Hospital of Hunan Province, The Hospital of Hunan University of Chinese Medicine, Changsha, China; ^5^Clinical Diagnosis and Therapy Center for Gliomas of Xiangya Hospital, Central South University, Changsha, China; ^6^ National Clinical Research Center for Geriatric Disorders, Xiangya Hospital, Central South University, Changsha, China

**Keywords:** gliomas, immune checkpoint genes, immunosuppressive, chemotherapy, CXCL

## Abstract

Gliomas are a type of malignant central nervous system tumor with poor prognosis. Molecular biomarkers of gliomas can predict glioma patient’s clinical outcome, but their limitations are also emerging. C-X-C motif chemokine ligand family plays a critical role in shaping tumor immune landscape and modulating tumor progression, but its role in gliomas is elusive. In this work, samples of TCGA were treated as the training cohort, and as for validation cohort, two CGGA datasets, four datasets from GEO database, and our own clinical samples were enrolled. Consensus clustering analysis was first introduced to classify samples based on CXCL expression profile, and the support vector machine was applied to construct the cluster model in validation cohort based on training cohort. Next, the elastic net analysis was applied to calculate the risk score of each sample based on CXCL expression. High-risk samples associated with more malignant clinical features, worse survival outcome, and more complicated immune landscape than low-risk samples. Besides, higher immune checkpoint gene expression was also noticed in high-risk samples, suggesting CXCL may participate in tumor evasion from immune surveillance. Notably, high-risk samples also manifested higher chemotherapy resistance than low-risk samples. Therefore, we predicted potential compounds that target high-risk samples. Two novel drugs, LCL-161 and ADZ5582, were firstly identified as gliomas’ potential compounds, and five compounds from PubChem database were filtered out. Taken together, we constructed a prognostic model based on CXCL expression, and predicted that CXCL may affect tumor progression by modulating tumor immune landscape and tumor immune escape. Novel potential compounds were also proposed, which may improve malignant glioma prognosis.

## Introduction

A glioma, accounting for 81% of malignant brain tumors, is a primary brain tumor originating from glial stem cells or progenitor cells (1, 2). The malignancy of gliomas is attributed to its rapid cell proliferation and abnormal angiogenesis (3). World Health Organization graded gliomas from I to IV based on tumor histological features. Grade IV gliomas, also known as GBM, progress aggressively, show high recurrence rate, and are resistant to tumor treatment, along with median survival time less than 14.6 months. Molecular biomarkers like IDH status, MGMT promoter status, 1p19q, ARTX have been considered as glioma progression-associated marker. Molecular classification like mesenchymal, classical, and proneural was also proposed to assist in predicting glioma progression. Current standard treatment for gliomas includes maximal surgical removal combined with radio-chemotherapy, but patients’ survival outcome is still unsatisfactory (4). Therefore, the exploration of potential biomarkers may benefit glioma patients and assist in clinical treatment decision.

Tumor microenvironment (TME) consists of tumor cells and non-tumor cells, such as microglia, peripheral macrophages, myeloid-derived suppressor cells, vascular endothelial cells, and others (5, 6). The infiltration of those non-tumor cells has been proven to modulate tumor progression and tumor treatment sensitivity. Therefore, chemokines secreted by gliomas affect not only TME components but also tumor progression (7).

The C-X-C motif chemokine ligand (CXCL) family widely participates in immunocyte recruitment and affecting tumor progression like tumor migration and angiogenesis (8, 9). For instance, CXCL1 promotes tumor angiogenesis in ovarian cancer (10). CXCL1, CXCL2, and CXCL8 participate in the formation of endothelial tube in cervical cancer (11). CXCL1, CXCL5, and CXCL16 are able to facilitate tumor metastasis like lung cancer (12) and gastric cancer (13). In gliomas, abnormal expression profiles of CXCL9, CXCL10, CXCL11, and CXCL12 were noticed among different pathological grade gliomas, implying their potential relationship with glioma progression (14–16). Nevertheless, their specific role in gliomas is elusive.

In our study, samples of TCGA were set as training cohort, while CGGA2, CGGA1, four datasets from GEO database, and our own clinical samples were treated as validation cohort (**Supplementary Figure 1A**). The expression profile of the CXCL family was first depicted. Then, consensus clustering analysis was introduced to establish the cluster model based on CXCL expression profile. Abnormal CXCL expression was noticed within the cluster model implying their role in glioma progression. In order to identify the main contributor of glioma progression and enhance the cluster model prognosis prediction accuracy, the elastic net analysis was introduced and calculated risk score of each sample. CXCL9, CXCL10, CXCL11, CXCL12, and CXCL14 were filtered as main contributors of glioma progression. High-risk samples exhibited worse clinical outcome, indicating the prognostic prediction ability of the risk model. Besides, high immunocyte infiltration ratio and immune checkpoint gene (ICG) expression were also discovered in high-risk samples. Therefore, potential compounds targeted to high-risk samples were also predicted.

## Results

### Abnormal Expression Profile of the CXCL Family May Affect Glioma Progression

**Figure 1:**
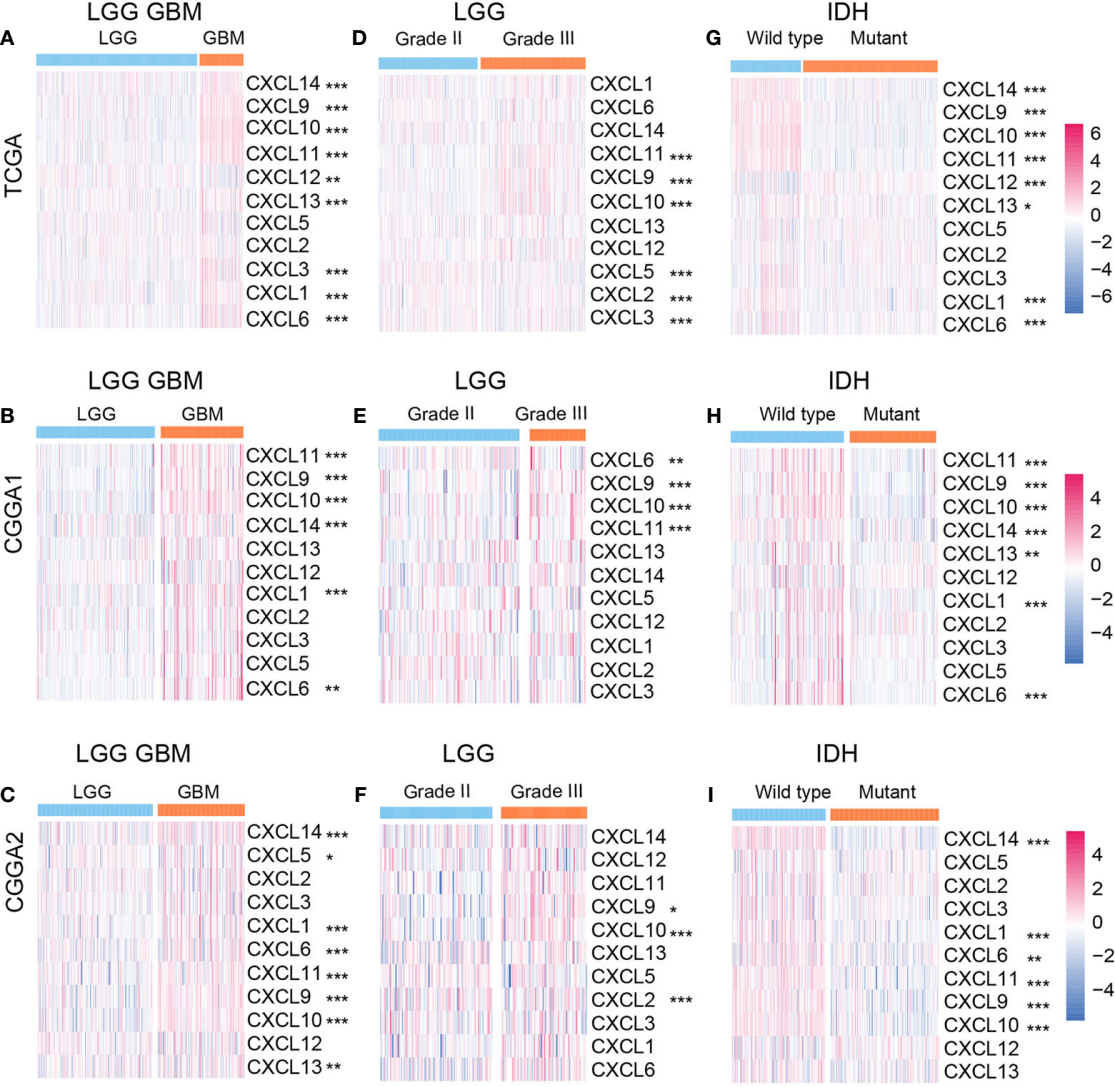
Relationships between CXCL expression patterns and clinical characters of gliomas. The expression profile of the CXCL family in the LGGGBM group **(A)** from the TCGA database and the validation cohort **(B, C)**. The heatmap of the CXCL family expression in LGG group **(D–F)** and GBM group **(G–I)** from the training and validation cohorts. *p < 0.05, **p < 0.01, ***p < 0.001.

We first explored the expression profile of members of the CXCL family. As illustrated, the mRNA expression levels of CXCL14 (P value < 0.001), CXCL9 (P value < 0.001), CXCL10 (P value < 0.001), CXCL11 (P value < 0.001), CXCL13 (P value < 0.001), CXCL3 (P value < 0.001), CXCL1 (P value < 0.001), CXCL6 (P value < 0.001) in the GBM group were significantly higher than that in the lower-grade gliomas (LGG) group, while CXCL12 (P value < 0.01) had a higher expression in the LGG group (**Figure 1A**) according to the training cohort. In the validation cohort, similar expression alternation was observed on CXCL11 (P value < 0.001), CXCL9 (P value < 0.001), CXCL10 (P value < 0.001), but not for CXCL12 and CXCL5 (**Figures 1B** **, C**). In LGG, the expressions of CXCL11 (P value < 0.001), CXCL9 (P value < 0.001), and CXCL10 (P value < 0.001) were higher in the Grade III group, and higher CXCL5 (P value < 0.001), CXCL2 (P value < 0.001), and CXCL3 (P value < 0.001) expression was noticed in the Grade II group (**Figure 1D**). Members like CXCL9 (P value < 0.001), CXCL10 (P value < 0.001) from the validation cohort manifested similar expression profile as that from the training cohort (**Figures 1E** **, F**). Members like CXCL2, CXCL3, CXCL6 in the validation cohort manifested different expression alternation comparing the results from TCGA dataset.

IDH status is a classical biomarker to predict the malignancy of gliomas, and mutant IDH gliomas showed better prognosis than IDH wild-type gliomas. Hence, we mapped the expression profile of the CXCL family based on IDH status. In the training cohort, CXCL14 (P value < 0.001), CXCL9 (P value < 0.05), CXCL10 (P value < 0.001), CXCL11 (P value < 0.001), CXCL6 (P value < 0.001), and CXCL1 (P value < 0.001) were enriched in the IDH wide-type gliomas (**Figure 1G**). Similar expression profile was also mapped in the validation cohort (**Figures 1H** **, I**). Together, abnormal expression of CXCLs may be associated with glioma progression.

### Cluster2 Samples Exhibit Aggressiveness Growth Pattern Than Cluster1 Samples

**Figure 2:**
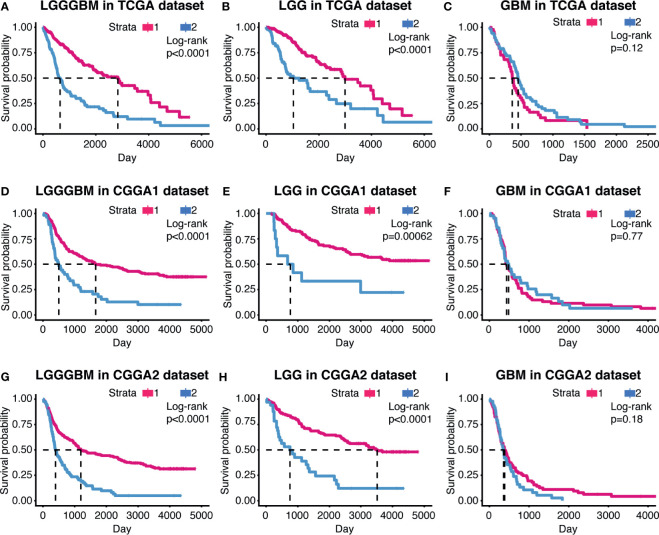
The prognostic value of the cluster model in gliomas. Kaplan–Meier survival analysis were used to show overall survival outcome difference between the two clusters in LGG, GBM, and LGGGBM samples from TCGA **(A–C)**, CGGA1 **(D–F)**, and CGGA2 **(G–I)**.

The samples in the TCGA dataset were divided into cluster1 and cluster2 (**Supplementary Figures 1B–E**). In the TCGA dataset, samples from cluster1 showed better overall survival (OS) in gliomas (p < 0.0001) and LGG (p < 0.0001) than samples from cluster2. Nevertheless, there were no significant survival outcome differences noticed in GBM, which may constrain from its population (p = 0.12) (**Figures 2A** **–C**).

The support vector machine (SVM) was used to learn the characteristics of the two cluster samples and reconstruct the cluster model in validation cohort (**Supplementary Figure 1F**). Similar overall survival difference between cluster1 and cluster2 was obtained, suggesting the clustering model can predict glioma patients’ prognosis and indicated CXCL can affect glioma progression (**Figures 2D** **–I**).

### Constructing the Risk Model Based on Elastic Net Regression Analysis

**Figure 3:**
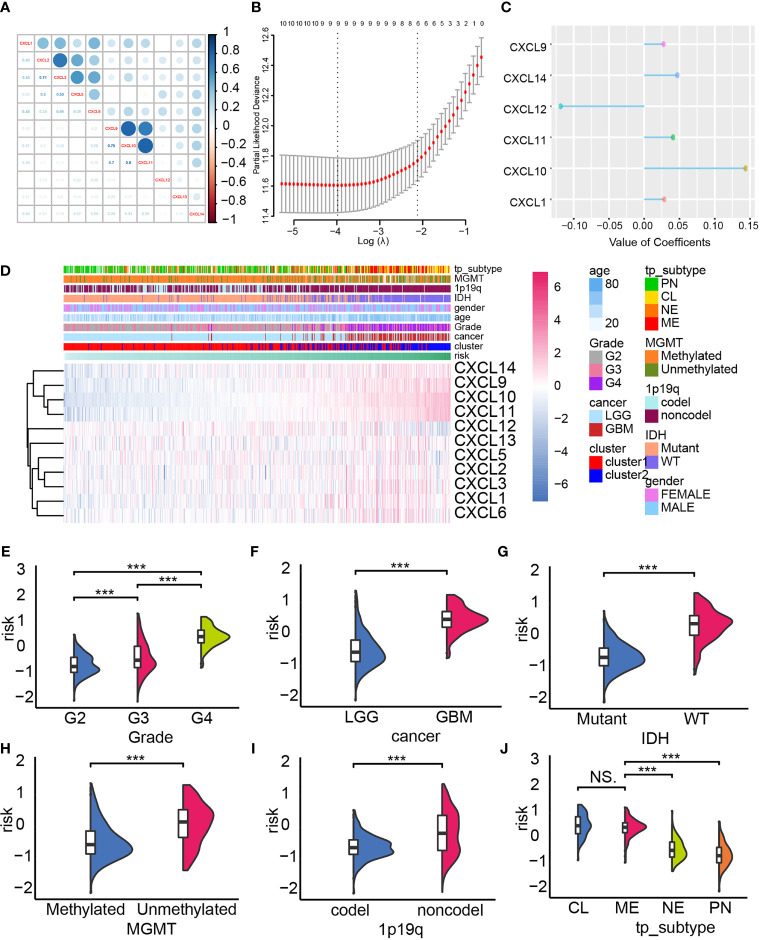
Constructing the risk model. **(A)** The co-expression network of the CXCL family. **(B, C)** The construction of the risk model based on the expression profile of the CXCL family by performing elastic net regression algorithm. **(D)** Heatmap displayed the alternation of the CXCL family members’ expression according to the risk model, and corresponding clinical features were also mapped. The distribution of the risk in gliomas’ pathological grade **(E)**, IDH status **(F)**, cancer type **(G)**, MGMG status **(H)**, 1p19q status **(I)**, and subtype **(J)**. NS, not significant, ***p < 0.001.

Next, we built a risk model by employing the elastic regression analysis in order to identify the main contributor that affect glioma progression. As the correlogram showed, two co-expression clusters (CXCL1, CXCL2, CXCL3, CXLC5, and CXCL6; CXCL9, CXCL10, and CXCL11) can be mapped, implying they may share similar regulator or exert similar function (**Figure 3A**). The elastic net regression algorithm identified five members of the CXCL family as glioma prognostic-related biomarkers, and the risk of each sample was calculated according to their coefficient (**Figures 3B** **, C**). The expression of the CXCL family of samples of TCGA datasets, along with their clinical features, was displayed by heatmap, which was arranged by their risk score (**Figure 3D**). The expression level of CXCL9, CXCL10, CXCL11, and CXCL14 was positively correlated with risk, while CXCL12 was associated with low risk.

In the TCGA datasets, aggressiveness gliomas were more likely to be calculated with higher risk score (**Figures 3E** **, F**). Meanwhile, high-risk samples were also categorized as malignant glioma subtype like IDH wild-type gliomas (**Figure 3G**), MGMT unmethylated gliomas (**Figure 3H**), 1p19q non-codel gliomas (**Figure 3I**), and mesenchymal/classical gliomas (**Figure 3J**). Similar conclusion can also be obtained from the validation cohort (**Supplementary Figures 2A–J**). Therefore, the risk score of each sample associated with malignant gliomas’ clinical feature, implying its ability in predicting glioma prognosis.

**Figure 4:**
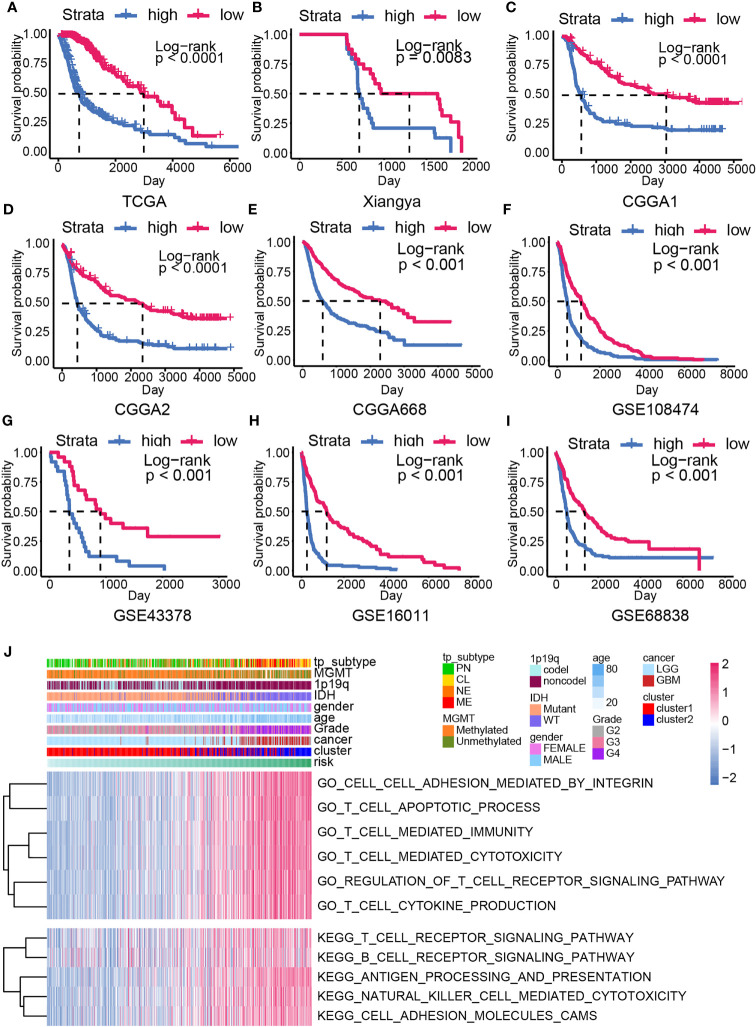
The overall survival analysis and biofunction prediction based on the risk model. Kaplan–Meier survival analysis based on the risk model from the training cohort **(A)** and the validation cohort, including Xiangya cohort **(B)**, CGGA1 **(C)**, CGGA2 **(D)**, CGGA688 **(E)**, GSE108474 **(F)**, GSE43378 **(G)**, GSE16011 **(H)**, GSE68838 **(I)**. **(J)** GO/KEGG enrichment analysis based on the GSVA analysis in the training cohort.

Overall survival analysis suggested worse clinical outcome in high-risk samples than low-risk samples in gliomas from TCGA dataset (**Figure 4A**). In our own data, the Xiangya cohort, high-risk samples possessed shorter median survival time than low-risk samples (**Figure 4B**, P = 0.0063). Similar results were also gained in other validation cohorts, including CGGA1 (**Figure 4C**, P < 0.001), CGGA2 (**Figure 4D**, P < 0.001), CGGA668 (**Figure 4E**, P < 0.001), GSE108474 (**Figure 4F**, P < 0.001), GSE43378 (**Figure 4G**, P = 0.00361), GSE16011 (**Figure 4H**, P < 0.001), GSE68838 (**Figure 4I**, P < 0.001). In LGG, high-risk samples also have shorter median survival time than low-risk samples, whereas no significant survival outcome difference was observed in the GBM (**Supplementary Figure 3**).

### High-Risk Samples Showed Higher Immunocyte Infiltration Ratio and ICGs Expression

To analyze the difference between high- and low-risk samples, the GO/KEGG enrichment analyses based on the GSVA analysis were conducted. Higher enrichment score of the immune-related pathways like T cell apoptotic process, T cell–mediated immunity, T cell–mediated cytotoxicity, regulation of T cell receptor signaling pathway, antigen processing and presentation, natural killer cell–mediated cytotoxicity, and cell adhesion–associated pathways were calculated in high-risk samples (**Figures 4J**). This result implied that CXCL may be able to modulate glioma progression by affecting immunocyte function.

**Figure 5:**
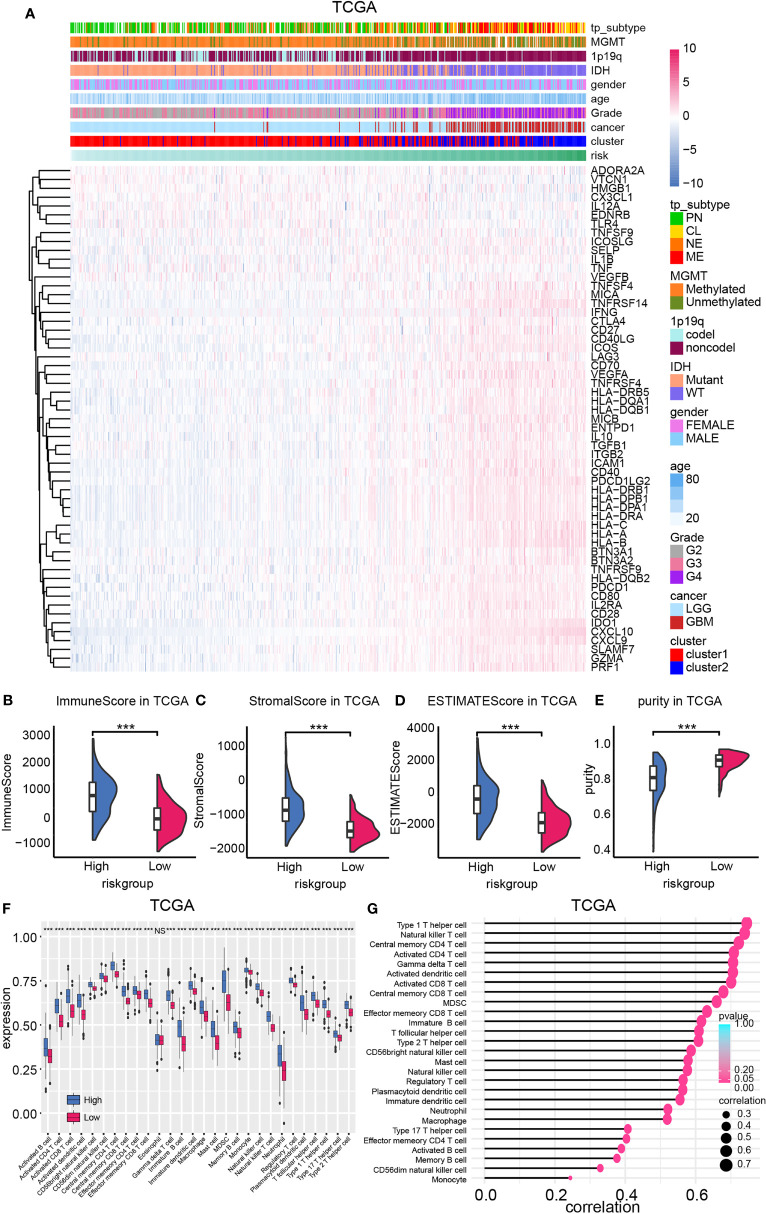
The expression of ICGs and immunocyte infiltration ratio. **(A)** ICG expression was mapped based on the risk model. The ImmuneScore **(B)**, StromalScore **(C)**, ESTIMATEscore **(D)**, and purity **(E)** difference between high- and low-risk group in TCGA database. **(F)** Immunocyte infiltration ratio enrichment score based on the risk model. **(G)** The correlation between the enrichment score of immunocytes and the risk model. NS, not significant, ***p < 0.001.

The expression profile of ICGs according to the risk score was mapped considering that the CXCL family plays a critical role in mediating immunocytes’ function. Positive correlation between ICG expression like HLAs, MICA, CD40LG, CD70, CD40, CTLA4, and samples’ risk was discovered (**Figure 5A**). Therefore, the microenvironment of high-risk samples may be immunosuppressed microenvironment. Then, immunocyte infiltration ratio was analyzed by conducting the ESTIMATE algorithm. The stromal score and immune score were higher in high-risk samples relative to low-risk samples, indicating the immune landscape of high-risk samples was more complicated (**Figures 5B–E**, **Supplementary Figures 4A, B**). Thereby, higher estimate score (the combination of stromal score and immune score) and lower tumor purity were also noticed in high-risk samples.

Immunocyte infiltration ratio was illustrated by employing the ssGSEA algorithm (**Figure 5F**, **Supplementary Figures 4C, D**). Results from both training cohort and validation cohort suggested that immunocytes like natural killer cells, T cells, B cells, macrophage were enriched in high-risk samples. Moreover, positive correlation between enrichment scores of immunocytes and the risk model was also noticed (**Figure 5G**, **Supplementary Figures 4E, F**). Together, high-risk samples infiltrated with more immunocytes and higher ICG expression, indicating its complicated, immunosuppressed microenvironment.

### Chemotherapy Suggestion Based on the Risk Model

**Figure 6:**
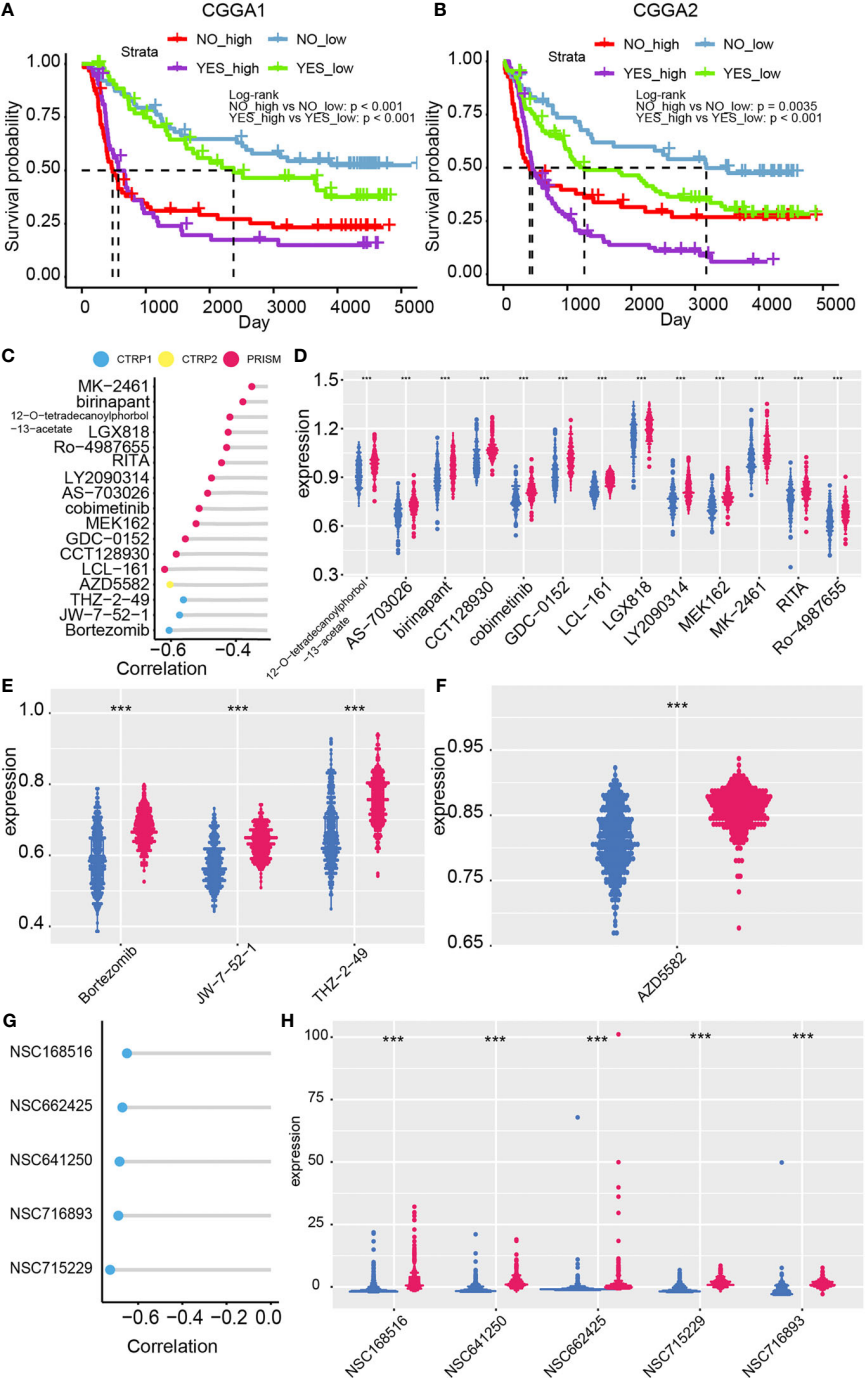
Chemotherapy suggestion based on the risk model. Chemotherapy efficacy difference between high- and low-risk group in the CGGA1 **(A)** and CGGA2 **(B)** datasets. **(C)** Correlation between risk and the AUC value of 17 candidate compounds. **(D–F)** Distribution of the AUC value of candidate compounds in the risk model. **(G)** Correlation between compounds’ IC50 and risk from Cellminer dataset. **(H)** The difference between IC50 of compounds and the risk model ***p < 0.001.

Temozolomide is the first-line drug for gliomas. Patients with high or low risk showed different sensitivity to temozolomide according to overall survival analysis (**Figures 6A, B**, P value < 0.001). Therefore, targeting to high-risk group by combining with other compounds may improve patients’ prognosis.

Potential sensitive drugs on high-risk glioma patients are predicted as previously reported, and 17 candidate compounds were identified (**Figure 6C**). Samples of lower AUC value of compounds indicated higher sensitivity to this compound. 12-O-tetradecanoylphorbol-13-acetate, AS-703026, birinapant, CCT128930, cobimetinib, GDC-0152, LCL-161, LGX818, LY2090314, MEK162, MK2461, RITA, and Ro-4987655 are identified from the PRISM database (**Figure 6D**); Bortezomib, JW-7-52-1, and THZ-2-49 are filtered out from CTRP 1 database (**Figure 6E**). AZD5582 is filtered out from CTRP 2 database (**Figure 6F**). Notably, ro-4987655, AZD5582, and MK-2461 may serve as novel compounds to treating gliomas. Moreover, the sensitivity of compounds from PubChem database based on CellMiner website was performed (**Figures 6G** **, H**). NSC68516, NSC662425, NSC641205, NSC716893, NSC715229 were filtered out as high-risk glioma patients’ potential targeted compounds. Taken together, temozolomide in combination with these potential targeted compounds may slow glioma progression.

### The Application of the Risk Model in Clinical

**Figure 7:**
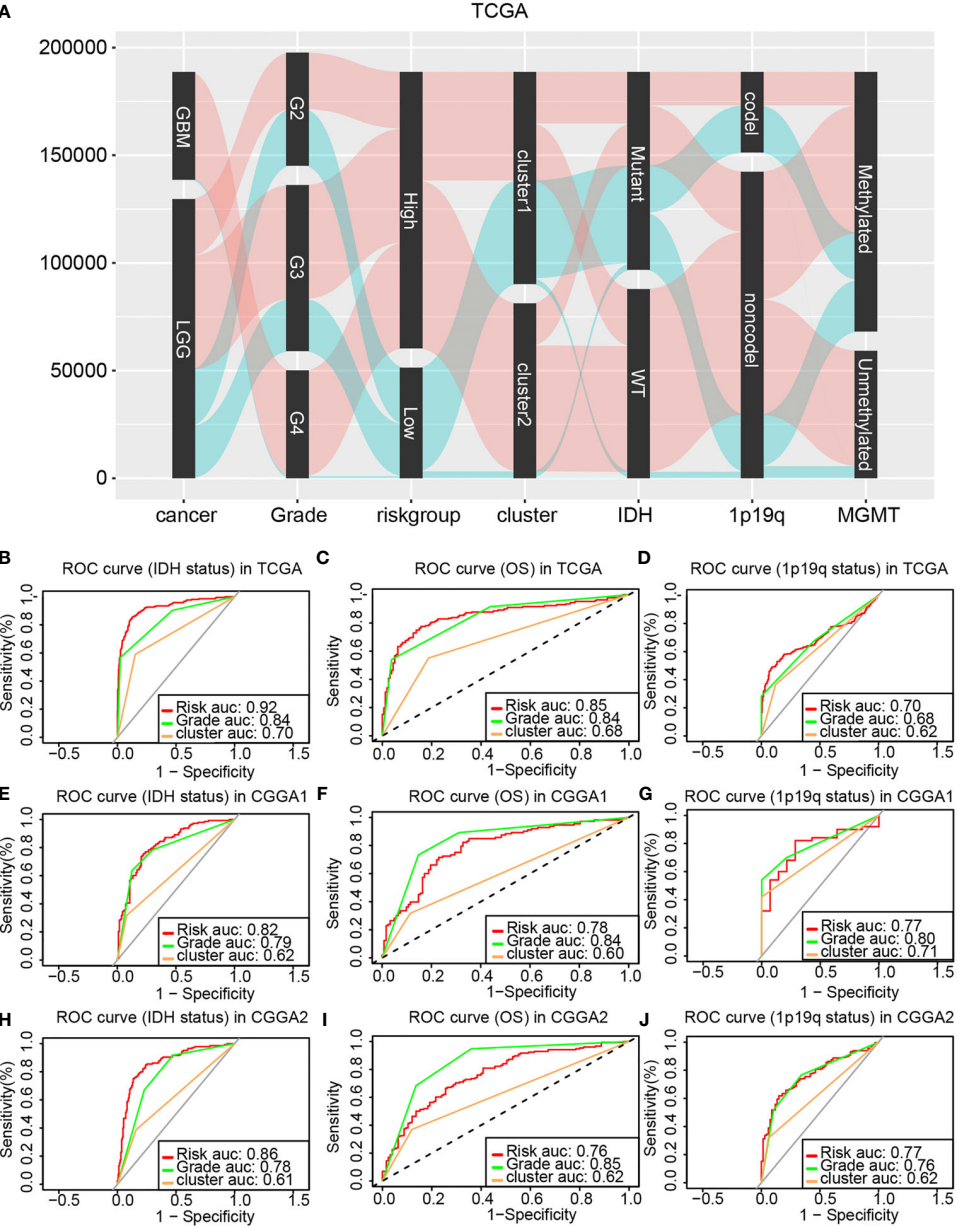
Comparing the prognostic ability of the cluster model, the risk model, and glioma pathological grade. **(A)** The Sankey diagram revealed the potential connection between glioma pathological grade, risk, cluster, IDH status, 1p19q status, and MGMT status. ROC curve generated based on the risk model by taking the IDH status **(B)**, OS **(C)**, and 1p19q status **(D)** as outcome variable in the training cohort. The validation of ROC curve in the CGGA1 **(E–G)** and CGGA2 **(H–J)** database.

The correlation between the risk model, the cluster model, tumor grade, IDH status, 1p19q status, and MGMT status was analyzed and displayed by the Sankey diagram. The Sankey diagrams showed that glioma patients in high-risk group were related to higher-grade gliomas, IDH wild type, 1p19q non-codeletion, and cluster 2 (**Figure 7A**). Therefore, both the cluster model and the risk model can predict glioma progression. Then, the ROC curves were used to compare the prognostic ability of the risk model, the glioma histological grade, and the cluster model when taking 1p19q codel status, IDH status, and OS as different outcome variable. Results from the training cohort and validation cohort suggested that the risk model as a better prognostic predictor than the cluster model, and as efficiency as tumor pathological grade (**Figures 7B** **–J**).

**Figure 8:**
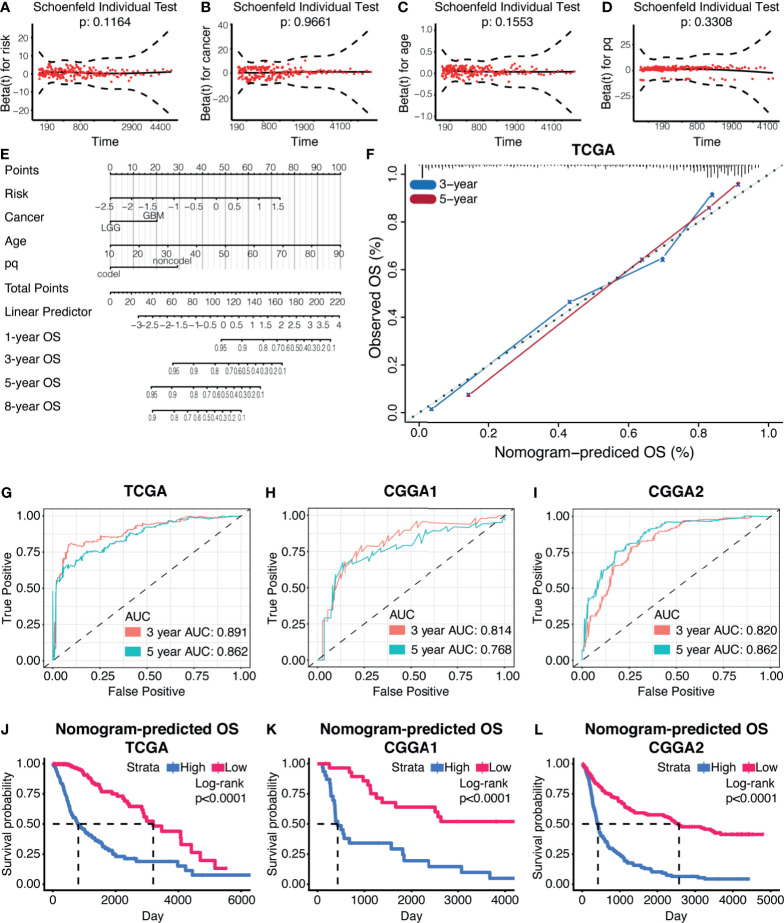
Prognostic nomogram based on the risk model. **(A–D)** The Schoenfeld test of the factors involved in the construction of the nomogram. **(E)** The nomogram based on the risk model. **(F)** The calibration curve of 3-year and 5-year OS based on the nomogram. ROC curves and AUC values from the nomogram of 3-year and 5-year OS, in TCGA datasets **(G)**, CGGA1 datasets **(H)**, and CGGA2 datasets **(I)**. **(J–L)** Survival analysis based on the nomogram in TCGA datasets **(J)**, CGGA1 datasets **(K)**, and CGGA2 datasets **(L)**.

Univariate Cox regression and multivariate Cox regression were first analyzed to identify glioma prognosis-associated clinical features. Risk (TCGA p<0.001, HR = 4.698), age (TCGA p <0.001, HR = 1.064), IDH (TCGA p <0.001, HR = 9.754), cancer (TCGA p<0.001, HR=8.750), and 1p19q (TCGA p <0.001, HR = 4.475) were considered as independent prognostic indexes for OS time (**Supplementary Figure 5**). Therefore, those factors were further used to construct a nomogram. The global Schoenfeld test suggested risk, cancer, age, and 1p19q were qualified factors for the construction of a nomogram as previously reported (**Figures 8A** **–D**).

Each variable was assigned a score, and the total points can be applied to predict the patient’s survival outcome (**Figure 8E**). The accuracy of the nomogram was verified by generating the calibration curves (**Figure 8F**). The ROC curve and AUC values of this statistics in predicting 3-year and 5-year OS of gliomas patients in TCGA were 0.891 and 0.862, respectively (**Figure 8G**). In the validation cohort, it was found that the ROC and AUC of the OS predicted at 3 and 5 years were 0.814 and 0.768 in CGGA1 while 0.820 and 0.862 in CGGA2, respectively (**Figures 8H** **, I**). The survival outcome difference between high- and low-risk groups were observed in the training and validation cohorts (**Figures 8J** **–L**). Together, the nomogram constructed by the risk model showed high accuracy in predicting glioma prognosis and can be applied to clinical.

## Discussion

Multiple studies reported that the CXCL family played a critical role in tumorigenesis, tumor cell proliferation and metastasis, tumor cell resistance to drugs, and tumor angiogenesis (17–20). For instance, CXCL14 promoted GBM progression by modulating tumor cell proliferation and migration (21). The STAT3 inhibitor can target the central nervous system tumor by induing immunocyte tumor homing in a CXCL10-dependent manner (22). CXCL12 was valued as a prognostic biomarker of LGG (23). In this work, CXCL9, CXCL10, and CXCL14 were also identified as potential vital regulators of glioma progression among the CXCL family.

The immune evasion mechanism of gliomas plays an important role in glioma tumor resistance to treatment and tumorigenesis (24–26). Biofunction prediction suggested that the CXCL family may promote glioma progression through inducing immune escape and affecting immunocyte infiltration. CD70 is a critical mediator of immunocytes’ activation in the tumor microenvironment (27, 28). High expression of classical ICGs like CTLA4 was also noticed in high-risk glioma samples, implying its immunosuppressive microenvironment (29).

Immunocytes like CD4 T cell, CD8 T cell, activated T cell, and memory T cell were preferentially infiltrated in high-risk samples. High immunocyte infiltration usually suggests high immunogenicity. However, a previous study reported that high ICG expression and function-impaired T cells in GBM together contribute to an immunosuppressed tumor microenvironment (30). Therefore, ICGs interfered in immunocytes’ function in high-risk samples, in the end resulting in tumor evasion from immune surveillance. For instance, PD-1 can inhibit immunocytes’ biofunction in gliomas (29, 31). CTLA-4 facilitates immunosuppressive microenvironment by inhibiting antigen-specific T cell activation and enhancing myeloid-derived suppressor cells (32, 33). Together, considering the CXCL family can induce immunosuppressive microenvironment and immunocyte infiltration in solid tumor (34–36), targeting the members of CXCL family may improve tumor immunotherapy efficacy.

A previous study reported that the CXCL family participated in gliomas’ resistance to chemotherapy (37), and similar conclusion can be obtained from our study. Therefore, novel compounds targeted to high-risk samples may improve that situation. Novel high-risk sample-targeted therapeutic drugs like Ro-4987655, AZD5582, and MK-2461 have been reported to play a role in other tumors, but not in gliomas. For example, Ro-4987655, a highly selective mitogen-activated protein kinase kinase (MEK) inhibitor (38), has proved its efficacy in BRAF V600 mutant melanoma, BRAF wild-type melanoma, and KRAS mutant NSCLC patients (39). AZD5582 can trigger cell apoptosis in pancreatic cancer cells (40) and non-small-cell lung cancer (41). MK-2461 is an ATP-competitive multitargeted inhibitor of activated c-Met and able to slow the progression of pancreatic cancer (42, 43). Additionally, compounds from PubChem database, like NSC168516 and NSC715229, were also predicted as high-risk glioma sample–sensitive drugs. Taken together, glioma patients’ survival time may be able to be prolonged by combining those compounds with temozolomide.

In conclusion, our research revealed the expression characteristics of the CXCL gene family and constructed a high-precision glioma prognosis model. Moreover, this model highlighted the relationship between CXCL and tumor immunogenicity and offered novel treatment strategies.

## Materials and Methods

### Data Processing

RNA‐seq data and corresponding clinical information of gliomas were obtained from the TCGA, CGGA [mRNAarray_301 (CGGA1) dataset, mRNAseq_325 (CGGA2) dataset, and mRNAseq_693 (CGGA668) dataset], and GEO database (GSE108474, GSE43378, GSE16011, and GSE68838). All expression profiles were transformed into log2(TPM+1).

For each glioma patient of the 50 samples, major exclusion criteria were incomplete follow-up data, poor quality of samples, and missing baseline clinicopathological features. Formalin-fixed paraffin-embedded tumor tissues were then collected for sequencing. One μg RNA per sample was used as input material for RNA sample preparations, and DNA was extracted and sheared followed by sequencing library preparation using NEBNext UltraTM RNA Library Prep Kit. Subsequently, PCR was performed with Phusion High-Fidelity DNA polymerase, Universal PCR primers, and the Index (X) Primer. Biotin-labeled probe was applied to capture target regions after removing the PCR primer. The captured libraries were sequenced on an Illumina Hiseq platform, and 125 bp/150 bp paired-end reads were generated. Raw data (raw reads) of fastq format were first processed through in-house perlscripts. In this step, clean data (clean reads) were obtained by removing reads containing adapter, ploy-N, and low-quality reads from raw data. Meanwhile, calculation of Q20, Q30, and GC content of the clean data were performed. All downstream analyses were based on clean data with high quality. Reference genome and gene model annotation files were downloaded from the genome website directly. The reference genome index was built using Hisat2 v2.0.5, and paired-end clean reads were aligned to the reference genome using Hisat2 v2.0.5, and Hisat2 was selected as the mapping tool. FeatureCounts v1.5.0-p3 was then applied to count the reads’ numbers in order to map to each gene. The TPM of each gene was calculated based on the gene length and reads count mapped to this gene. Glioma sample collection was approved by the ethics committee of Xiangya Hospital.

### Construction of Prognostic Model

By using the R package “Consensus Cluster Plus” to perform consensus cluster analysis, the samples were divided into different groups to create a clustering model (44, 45). The optimum amount of Clusters was decided according to the cumulative distribution function plots and consensus matrices (46).

Support vector machine was introduced to reconstruct the cluster model in the validation cohort based on the characteristic of the cluster model with R package “e1071.” Kernel of algorithm was set as radial. Self-validation was conducted with R package “caret,” and the sensitivity of the cluster model was 0.9888 and the specificity was 0.9795 (range of sensitivity and specificity is 0 to 1).

The elastic net regression algorithm and their coefficient were calculated automatically (47). A risk system was established based on gene coefficients. According to the median of risk, patients were divided into high- and low-risk groups. Risk score is calculated as follows:

Risk = 0.028 * CXCL1 + 0.027 * CXCL9 + 0.14 * CXCL10 + 0.04 * CXCL11 + (−0.12 * CXCL12) + 0.047 * CXCL14

### Biological Function Prediction

The GO and KEGG analyses were carried out based on GSVA analysis, and corresponding information was downloaded from the molecular signature database (MSigDB) (48, 49). Results with false discovery rate <0.05 were considered as significant (50, 51).

The ESTIMATE algorithm was applied to evaluate the composition of the tumor microenvironment (52). The immunocyte infiltration ratio was calculated by performing the ssGSEA algorithm as the previous study reported (53, 54).

### Potential Compounds Prediction

Information about drug sensitivity was downloaded from the Profiling Relative Inhibition Simultaneously in Mixtures (PRISM) and the Cancer Therapeutics Response Portal (CTRP) database. Cell line expression matrix was obtained from Cancer Cell Line Encyclopedia. R package “pRRophetic” was introduced to predict sample sensitivity to certain compounds, and lower AUC values represented higher sensitivity. The knn imputation strategy was applied to imputed NA value in the expression matrix, and we discarded samples with more than 30% NA value. The “limma” package was performed to identify potential drugs, and correlation <−0.7 was set as threshold (55). Similar strategy was applied to predict drug sensitivity of compounds from Cellminer database.

### Survival Analysis and Nomogram

Kaplan-Meier analysis was used to generate survival curves, and the validity was evaluated by log-rank test. The receiver operating characteristic (ROC) curve and the area under the curve (AUC, AUC range is 0 to 1) were introduced to compare the predictive capabilities of different models. Univariate and multivariate Cox regression analyses were used to filter prognostic variables (P‐value < 0.05). Therefore, these variables have been verified by Schoenfeld’s test to construct a nomogram with the R package “survival” and “RMS,” respectively (54, 56–59). The calibration curve and ROC were used to evaluate the accuracy of the nomogram for OS prediction.

### Statistical Analysis

Statistical analysis was performed by R (version 3.6.2). Wilcoxon rank‐sum test was used to compare two groups. One‐way ANOVA was used to compare multiple groups. Spearman correlation analysis was used for correlation analysis (60). NS, not statistically significant; *P < 0.05; **P < 0.01; ***P < 0.001. P‐value < 0.05 was considered as statistically significant.

## Data Availability Statement

The original contributions presented in the study are included in the article/**Supplementary Material**. Further inquiries can be directed to the corresponding authors.

## Author Contributions

ZW, YL, and YM: Writing—original draft, writing—review, editing, data curation, and formal analysis. HZ and ZD: Investigation and methodology. WY, HC, and XZ: Validation. ZL and QC: Conceptualization, supervision, project administration, and funding acquisition. All authors contributed to the article and approved the submitted version.

## Funding

This study is supported by the National Nature Science Foundation of China (NO. 82073893, NO. 81873635, NO. 81703622); the China Postdoctoral Science Foundation (NO.2018M633002); the Natural Science Foundation of Hunan Province (NO. 2018JJ3838, NO. 2018SK2101); the Hunan Provincial Health and Health Committee Foundation of China (C2019186); and Xiangya Hospital Central South University postdoctoral foundation, and the Fundamental Research Funds for the Central Universities of Central South University (No. 2021zzts1027).

## Conflict of Interest

The authors declare that the research was conducted in the absence of any commercial or financial relationships that could be construed as a potential conflict of interest.

## Publisher’s Note

All claims expressed in this article are solely those of the authors and do not necessarily represent those of their affiliated organizations, or those of the publisher, the editors and the reviewers. Any product that may be evaluated in this article, or claim that may be made by its manufacturer, is not guaranteed or endorsed by the publisher.
