# Supplementary material for: The CXCL Family Contributes to Immunosuppressive Microenvironment in Gliomas and Assists in Gliomas Chemotherapy
Source: Front Immunol. 2021 Sep 13;12:731751. doi: 10.3389/fimmu.2021.731751 (PMC8482424; doi:10.3389/fimmu.2021.731751)
Supplement: Supplementary file 1 [file DataSheet_1.pdf]

Supplementary figure legend

Supplementary figure 1. The flow chart and the construction of the cluster model. (A) The flow chart of this study. (B-D) The construction of the cluster model. (E) The PCA diagram of the cluster model. (F) Characteristic of the cluster model learning by employing support vector machine algorithm.

Supplementary figure 2. The distribution of the risk model. The distribution of the risk model in gliomas pathological grade (A-B), cancer type (C-D), IDH status (E-F), 1p19q status (G-H), MGMT status (I-J) in the CGGA1 and CGGA2 cohort. NS, not significant, **p <0.01, ***p <0.001.

Supplementary figure 3. The OS analysis in LGG and GBM. The survival outcome difference between high and low risk group from the LGG and GBM group in the TCGA (A-B), CGGA1 (C-D) and CGGA2 datasets (E-F).

Supplementary figure 4. Results from ESTIMATE algorithm and immunocytes infiltration ratio in validation cohort. The distribution of IMMUNEscore, STROMALscore, ESTIMATEscore and purity in high and low risk group in CGGA1 (A) and CGGA2 (B) dataset. Immunocytes infiltration ratio in CGGA1 (C) and CGGA2 (D) dataset. The correlation between immunocytes enrichment score and risk in CGGA1 (E) and CGGA2 (F) dataset. NS, not significant, *p <0.05, **p <0.01, ***p <0.001.

Supplementary figure 5. Univariate and multivariate Cox regression. Univariate and multivariate Cox regression in the TCGA (A-B), CGGA1 (C-D), CGGA2 (E-F) dataset.

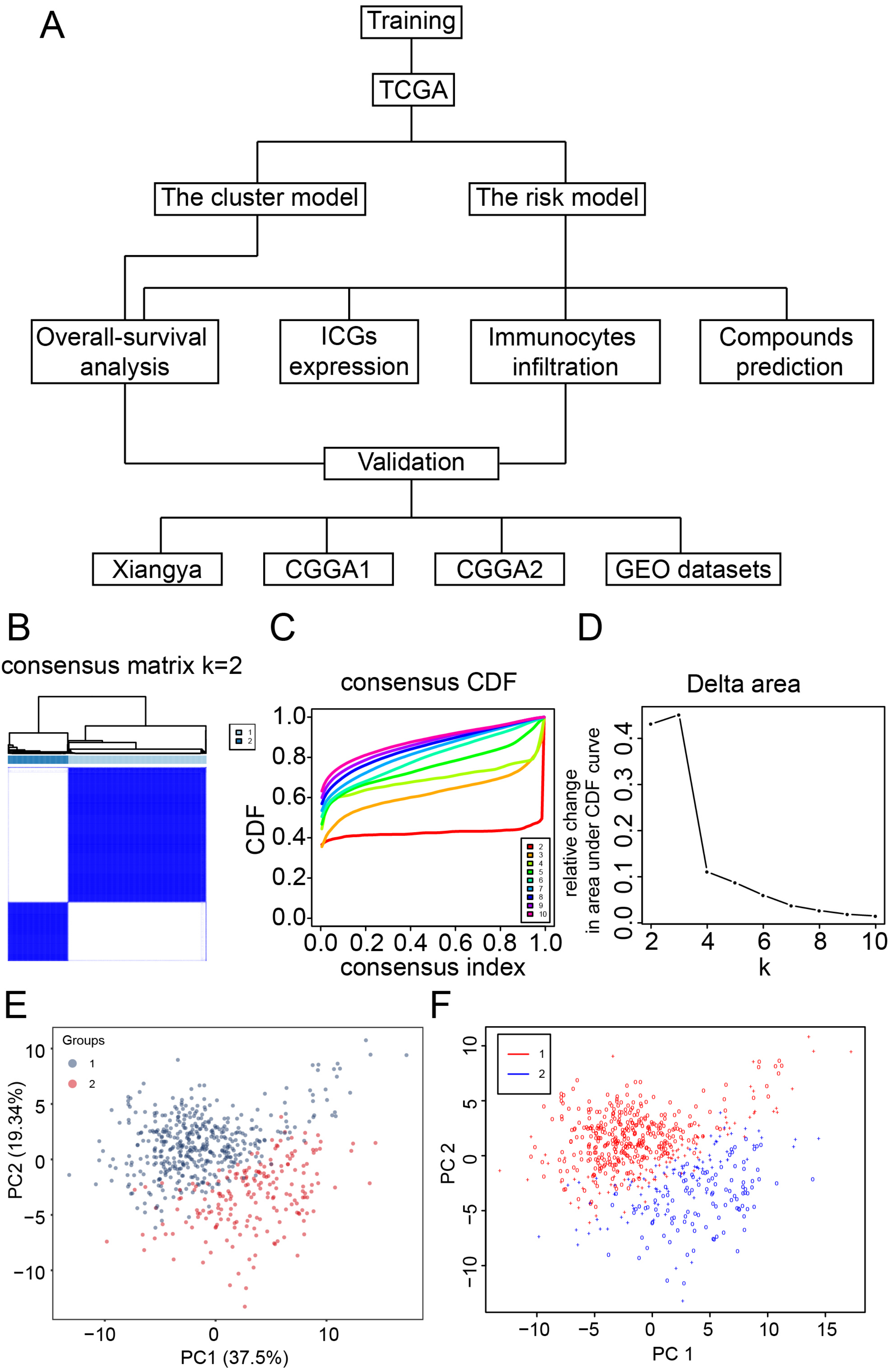
A
Training
TCGA
The cluster model
The risk model
Overall-survival analysis
ICGs expression
Immunocytes infiltration
Compounds prediction
Validation
Xiangya
CGGA1
CGGA2
GEO datasets
B
consensus matrix k=2
C
consensus CDF
CDF
consensus index
D
Delta area
relative change in area under CDF curve
k
E
Groups
PC2 (19.34%)
PC1 (37.5%)
F
PC 2
PC 1

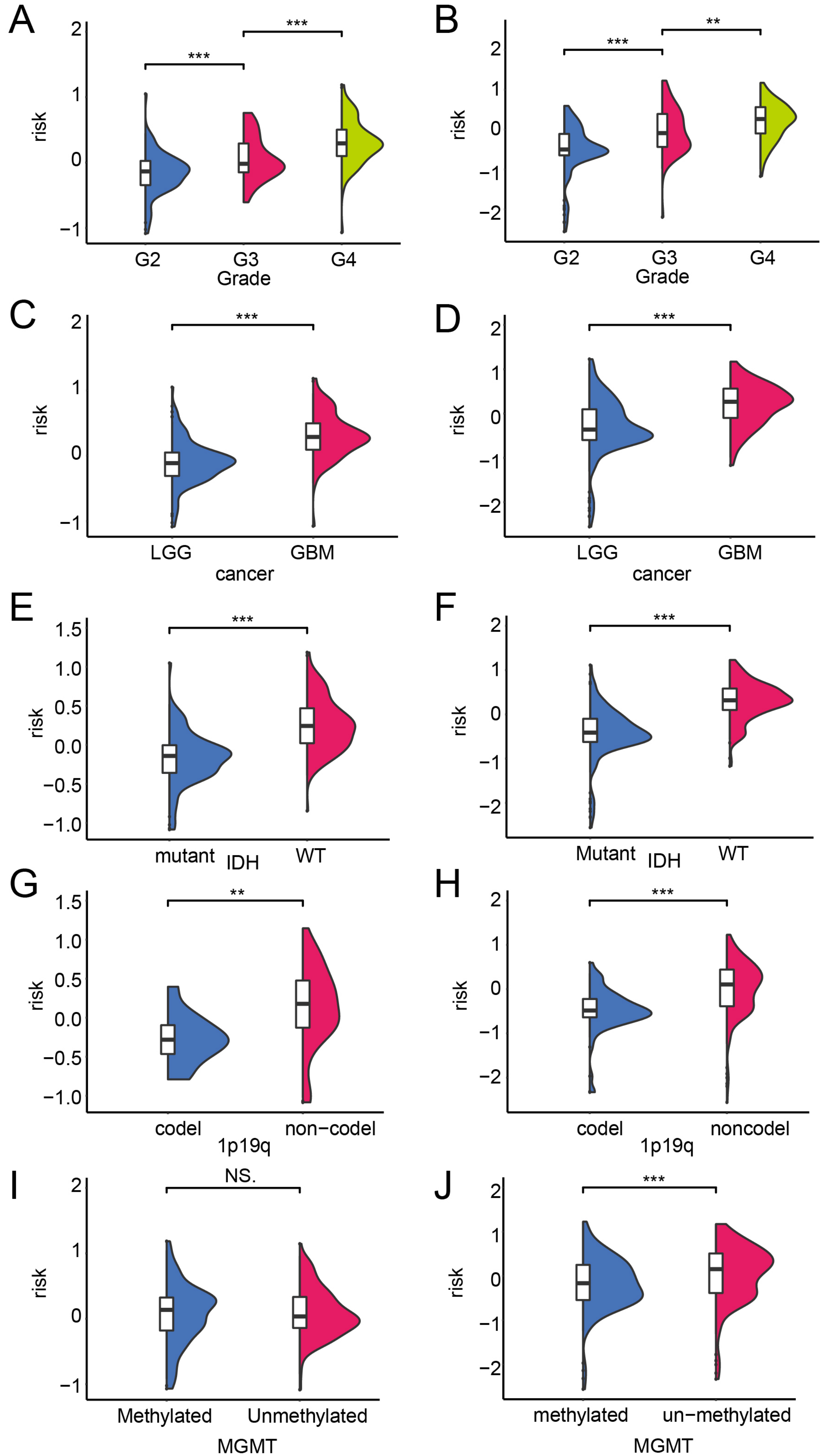

A
B
C
D
E
F
G
H
I
J
risk
Grade
G2
G3
G4
***
**
cancer
LGG
GBM
IDH
mutant
Mutant
WT
1p19q
codel
non−codel
noncodel
MGMT
Methylated
Unmethylated
methylated
un−methylated
NS.

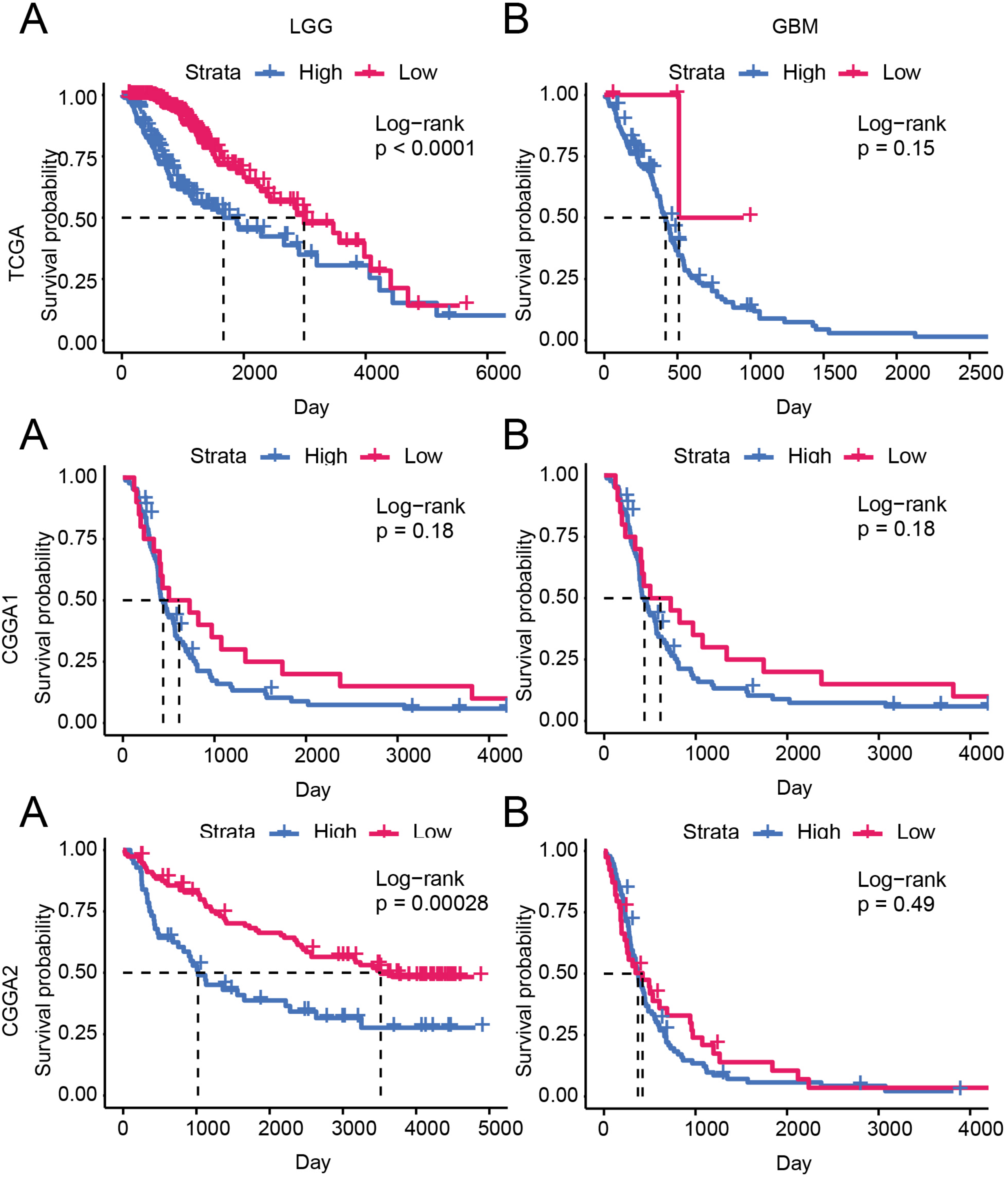

A
LGG
Strata High Low
Log−rank
p < 0.0001
TCGA
Survival probability
Day
B
GBM
Strata High Low
Log−rank
p = 0.15
Survival probability
Day
A
Strata High Low
Log−rank
p = 0.18
CGGA1
Survival probability
Day
B
Strata High Low
Log−rank
p = 0.18
Survival probability
Day
A
Strata High Low
Log−rank
p = 0.00028
CGGA2
Survival probability
Day
B
Strata High Low
Log−rank
p = 0.49
Survival probability
Day

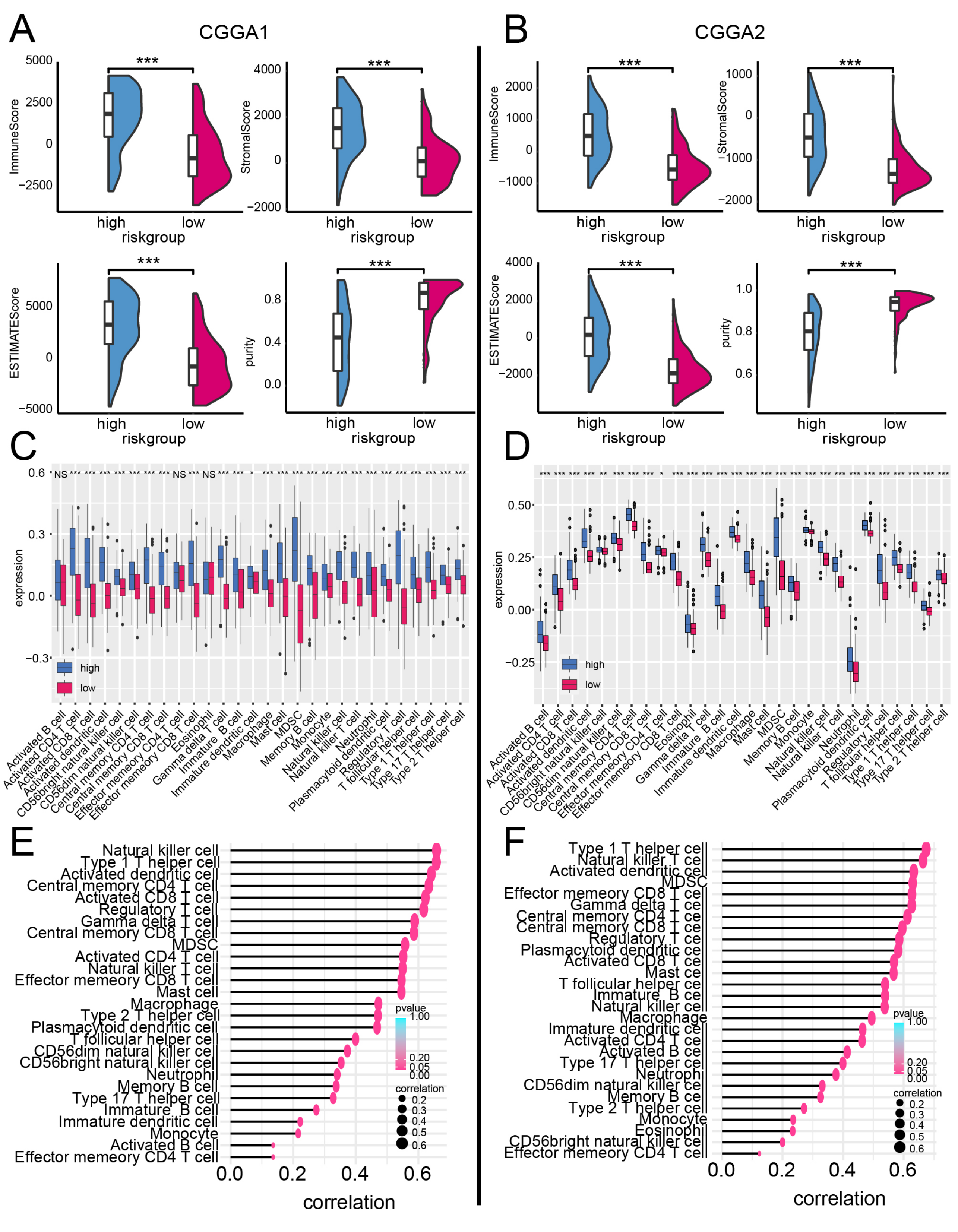
A
CGGA1
B
CGGA2
ImmuneScore
StromalScore
ESTIMATEScore
purity
riskgroup
high
low
***
C
D
expression
NS
high
low
Activated B cell
Activated CD4 T cell
Activated CD8 T cell
Activated dendritic cell
CD56bright natural killer cell
CD56dim natural killer cell
Central memory CD4 T cell
Central memory CD8 T cell
Effector memeory CD4 T cell
Effector memeory CD8 T cell
Eosinophil
Gamma delta T cell
Immature B cell
Immature dendritic cell
Macrophage
Mast cell
MDSC
Memory B cell
Monocyte
Natural killer cell
Natural killer T cell
Neutrophil
Plasmacytoid dendritic cell
Regulatory T cell
T follicular helper cell
Type 1 T helper cell
Type 17 T helper cell
Type 2 T helper cell
E
F
pvalue
correlation

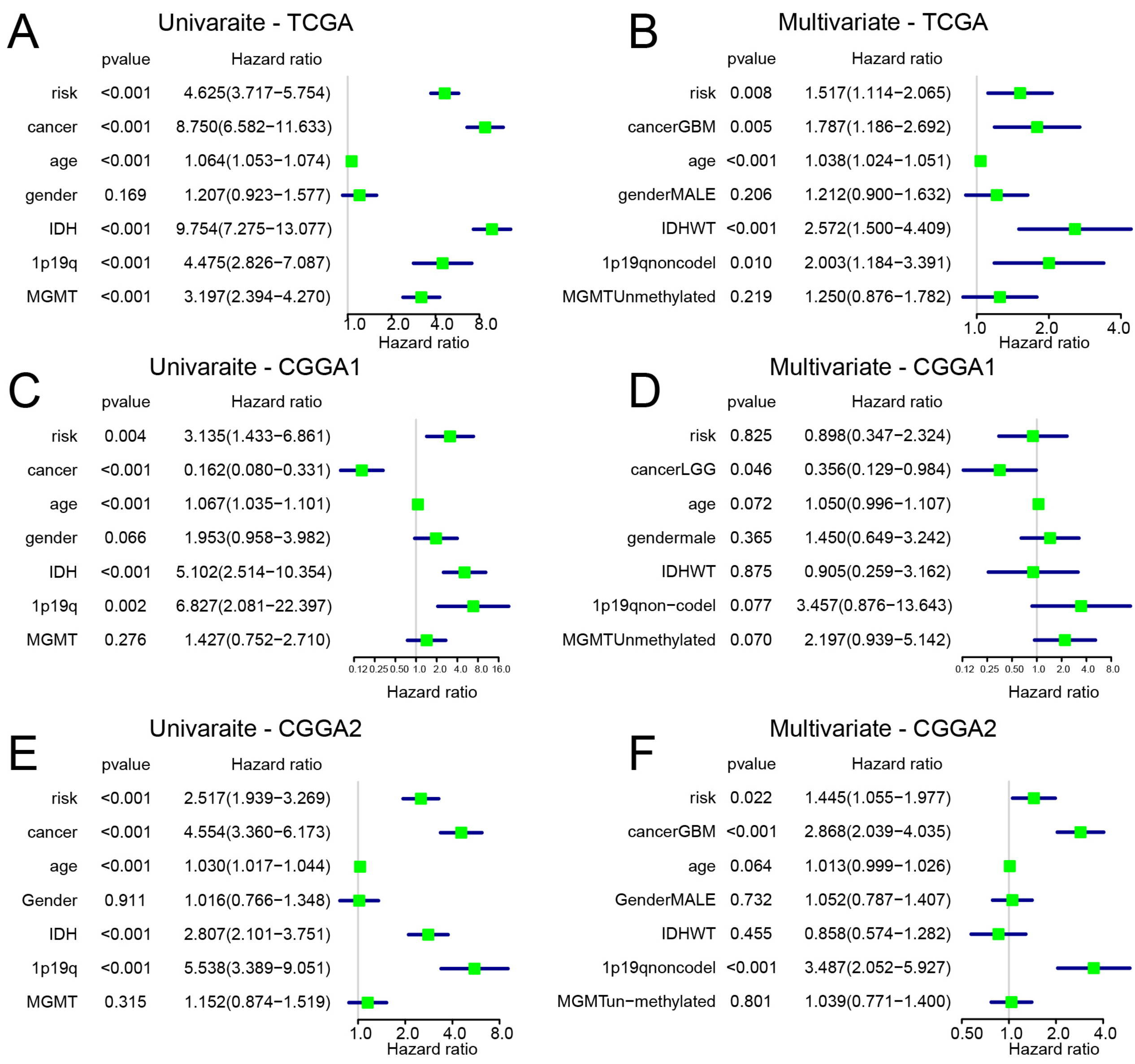
A
Univaraite - TCGA
pvalue
Hazard ratio
risk <0.001 4.625(3.717−5.754)
cancer <0.001 8.750(6.582−11.633)
age <0.001 1.064(1.053−1.074)
gender 0.169 1.207(0.923−1.577)
IDH <0.001 9.754(7.275−13.077)
1p19q <0.001 4.475(2.826−7.087)
MGMT <0.001 3.197(2.394−4.270)
1.0 2.0 4.0 8.0
Hazard ratio
B
Multivariate - TCGA
pvalue
Hazard ratio
risk 0.008 1.517(1.114−2.065)
cancerGBM 0.005 1.787(1.186−2.692)
age <0.001 1.038(1.024−1.051)
genderMALE 0.206 1.212(0.900−1.632)
IDHWT <0.001 2.572(1.500−4.409)
1p19qnoncodel 0.010 2.003(1.184−3.391)
MGMTUnmethylated 0.219 1.250(0.876−1.782)
1.0 2.0 4.0
Hazard ratio
C
Univaraite - CGGA1
pvalue
Hazard ratio
risk 0.004 3.135(1.433−6.861)
cancer <0.001 0.162(0.080−0.331)
age <0.001 1.067(1.035−1.101)
gender 0.066 1.953(0.958−3.982)
IDH <0.001 5.102(2.514−10.354)
1p19q 0.002 6.827(2.081−22.397)
MGMT 0.276 1.427(0.752−2.710)
0.12 0.25 0.50 1.0 2.0 4.0 8.0 16.0
Hazard ratio
D
Multivariate - CGGA1
pvalue
Hazard ratio
risk 0.825 0.898(0.347−2.324)
cancerLGG 0.046 0.356(0.129−0.984)
age 0.072 1.050(0.996−1.107)
gendermale 0.365 1.450(0.649−3.242)
IDHWT 0.875 0.905(0.259−3.162)
1p19qnon−codel 0.077 3.457(0.876−13.643)
MGMTUnmethylated 0.070 2.197(0.939−5.142)
0.12 0.25 0.50 1.0 2.0 4.0 8.0
Hazard ratio
E
Univaraite - CGGA2
pvalue
Hazard ratio
risk <0.001 2.517(1.939−3.269)
cancer <0.001 4.554(3.360−6.173)
age <0.001 1.030(1.017−1.044)
Gender 0.911 1.016(0.766−1.348)
IDH <0.001 2.807(2.101−3.751)
1p19q <0.001 5.538(3.389−9.051)
MGMT 0.315 1.152(0.874−1.519)
1.0 2.0 4.0 8.0
Hazard ratio
F
Multivariate - CGGA2
pvalue
Hazard ratio
risk 0.022 1.445(1.055−1.977)
cancerGBM <0.001 2.868(2.039−4.035)
age 0.064 1.013(0.999−1.026)
GenderMALE 0.732 1.052(0.787−1.407)
IDHWT 0.455 0.858(0.574−1.282)
1p19qnoncodel <0.001 3.487(2.052−5.927)
MGMTun−methylated 0.801 1.039(0.771−1.400)
0.50 1.0 2.0 4.0
Hazard ratio
